# 

**Published:** 1880-09

**Authors:** 


### BOOKS AND PAMPHLETS RECEIVED.

Pathogenetic Outlines of Homœopathic Drugs. By Carl Meingke, M.D.

Sea Air and Sea Bathing. By J. H. Packard, M.D. Philadelphia: P. Blakiston; 1880.

A Text Book of Physiology. By M. Foster, M.D., F.R.S. Philadelphia: H. C. Lea's Son & Co.; 1880.

The Black Arts in Medicine. By Jno. D. Jackson, M.D. Cincinnati: R. Clarke & Co.; 1880.

Clinical Treatise on Diseases of Nervous System. By M. Rosenthal, Vienna. Vol. II. New York: Wm. Wood & Co.; 1879.

The Practitioner's Reference Book. By R. J. Dunglison, M.D. Lindsay & Blakiston, Philadelphia; 1880.

Transactions American Gynæcological Society. Vol. IV, for year 1879. Boston: Houghton, Mifflin & Co.; 1880.

A Guide to the Practical Examination of Urine. By James Tyson, M.D. Philadelphia: Lindsay & Blakiston; 1880.

Health and Healthy Homes. By Geo. A. Wilson, M.D. Philadelphia: Lindsay & Blakiston; 1880.

Lessons in Gynæcology. By Wm. Goodell, M.D. Philadelphia: D. G. Brinton; 1880.

Transactions Indiana State Medical Society for 1880. Thirtieth Annual Session. Carlon & Hallenbeck, Indianapolis; 1880.

The Female Pelvic Organs. By H. Savage, M.D., London. New York: Wm. Wood & Co.; 1880.

Report of Michigan State Board of Health for year 1879. Lansing, Michigan; 1880.

Naso-Pharyngeal Catarrh. By M. F. Coomes, M.D. Louisville, Ky.: Bradley & Gilbert; 1880.

The Microscopist's Manual for 1879. Price 25c. Industrial Publication Company, New York; 1880.

Transactions of the Tenth Annual Session of Virginia Medical Society; 1879.

Relations of Communities and States during Epidemics. By Hon. James B Eustis New Orleans: 1880.

Time of Conception and Duration of Pregnancy. By Dr. Geo. J. Engleman. St. Louis; 1880.

Fluid Extracts for the Coming Pharmacopœia. Reprint from *Therapeutic Gazette*, April 15, 1880.

A Severe Case of Puerperal Eclampsia. E. Day, M.D.

Relation of the Medical Profession to the Trade Interests of Materia Medica, and a Note on Pepsin. Edw. Squibb, M,D.

Fracture of the Patella: A Study of 127 Cases. By F. H. Hamilton, M.D. Chas. L. Benningham & Co., New York; 1880.

The Management of Children in Sickness and in Health. By A. M. Hale, M.D. Philadelphia: P. Blakiston; 1880.

Post Mortem Examinations. By Prof. R. Virchow. Philadelphia: P. Blakiston; 1880.

Thos. Keith and Ovariotomy. By J. Marion Sims, M.D. New York: Wm. Wood & Co.; 1880.

Aids to Chemistry. Part II: Inorganic—The Metal. By C. E. A. Semple. New York: G. P. Putnam's Sons; 1880.

Perinephritis. By V. P. Gibney, M.D., New York.

Kolpo-Cystotomy by Electro-Cautery. By Jno. Byrne, M.D.

Cases of Tracheotomy for Croup and Diphtheria. By E. F. Ingals, M.D.

Diseased Meats and Foul Water. By H. M. Lyman, M.D.

The Bromide of Ethyl as an Anæsthetic. By J. Marion Sims, M.D., New York.

Aids to Materia Medica and Therapeutics. By C. E. A. Semple. New York: G. P. Putnam's Sons; 1880.

Aids to Chemistry. Part III: Organic. By C. E. A. Semple. New York: G. P. Putnam's Sons; 1880.

Aids to Physiology. By B. T. Lowne, F.R.C.S. New York: G. P. Putnam's Sons; 1880.

Aids to Chemistry. Part I: Inorganic—The Non-Metallic Elements. By C. E. A. Semple. New York: G. P. Putnam's Sons; 1880.

Transactions of the Medical Society of Tennessee; 47th Annual Meeting; 1880.

The Histology of the Blood Vessels. By E. C. Wendt, M.D. New York: D. Appleton & Co.

The Therapeutic Value of Iodide of Ethyl. By R. M. Lawrence, M.D.

The American Medical College Association; 4th Annual Meeting, New York, 5-31-80 and 6-1-80.

Physicians and their Patients. A Lecture by C. H. Merrick, M.D., Portland, Oregon.

Annual Announcement of University of City of New York for 1880-81.

Study of Fractures of Inferior Extremity. By L. S. Pilcher, M.D., Brooklyn, N. Y.

Indication for an Early Trepanation of the Mastoid Process in Acute Otitis, etc., etc. By F. C. Hotz, M.D.

Annual Announcement St. Paul Medical College.

Division of the Sphincter Ani Muscle as a Therapeutic Measure. By C. B. Kelsey, M.D., New York.

Optico-Ciliary Neurotomy. By J. J. Chisholm, M.D.

Thirteenth Annual Catalogue Detroit Medical College, 1880–81.

On Coccygodynia. By E. W. Jenks, M.D.

Thirty-eighth Annual Catalogue of Rush Medical College, 1880–81.

Report of N. O. A. S. Ass'n on Construction and Management of Privies, 1880.

Sea Sickness. By Geo. M. Beard, M.D.

Prolapse of the Ovaries. By Paul F. Mundé, M.D.

Announcement of Medical Department of University of Pennsylvania; 115th Annual Session, 1880-81.

Annual Announcement of Trinity Medical School, Toronto; Session 1880–81.

Chirurgie Antiseptique. Principes Modes d'Application et Resultats du Pansement de Lister. Par le Dr. Just Lucas, Championnière, Paris; 1880.

---

DR. LEVINSTEIN, of Berlin, Prussia, has had under treatment for morphinism eighty-two men and twenty-eight women; of these 110 patients, thirty-two were physicians, eight physicians' wives, seven persons more or less intimately connected with the profession, eight apothecaries, and one apothecary's wife. Of the thirty-two physicians, twenty-eight relapsed, as did all the apothecaries.—*Gaillard's Med. Jour.*

WE regret to announce the death of Dr. F. H. Davis of this city, the talented son of our senior editor, Dr. N. S. Davis, and we deeply sympathize with our associate in this, his great bereavement.

### ANNOUNCEMENTS FOR THE MONTH.

### SOCIETY MEETINGS.

Chicago Medical Society—Mondays, Sept. 6 and 20.
West Chicago Medical Society—Mondays, Sept. 13 and 27.
Biological Society—Wednesday, Sept. 1.

### CLINICS.

MONDAY.

Eye and Ear Infirmary—2 p. m., Ophthalmological, by Prof. Holmes; 3 p. m., Otological, by Prof. Jones.

Mercy Hospital—2 p. m., Surgical, by Prof. Andrews.

Rush Medical College—2 p. m., Dermatological and Venereal, by Prof. Hyde; 3 p. m., Medical, by Dr. Bridge.

Woman's Medical College—2 p. m., Dermatological and Venereal, by Prof. Maynard; 3 p. m., Diseases of the Chest, Prof. Ingals.

TUESDAY.

Cook County Hospital—2 to 4 p. m., Medical and Surgical Clinics.

Mercy Hospital—2 p. m., Medical, by Prof. Quine.

WEDNESDAY.

Chicago Medical College—2 p. m., Eye and Ear, by Prof. Jones.

Rush Medical College—3:30 to 4:30 p. m., Diseases of the Chest, by Dr. E. Fletcher Ingals.

THURSDAY.

Chicago Medical College—2 p. m., Gynæcological, by Prof. Jenks.

Rush Medical College—3 p. m., Diseases of the Nervous System, by Prof. Lyman.

Eye and Ear Infirmary—2 p. m., Ophthalmological, by Dr. Hotz.

Woman's Medical College—3 p. m., Surgical, by Prof. Owens.

FRIDAY.

Cook County Hospital—2 to 4 p. m., Medical and Surgical Clinics.

Mercy Hospital—2 p. m., Medical, by Prof. Davis.

SATURDAY.

Rush Medical College—2 p. m., Surgical, by Prof. Gunn.

Chicago Medical College—2 p. m., Surgical, by Prof. Isham; 3 p. m., Neurological, by Prof. Jewell.

Woman's Medical College—11 a. m., Ophthalmological, by Prof. Montgomery; 2 p. m., Gynæcological, by Prof. Fitch.

Daily Clinics, from 2 to 4 p. m., at the Central Free Dispensary, and at the South Side Dispensary.

